# LIMB-Q Kids—German Translation and Cultural Adaptation

**DOI:** 10.3390/children9091405

**Published:** 2022-09-16

**Authors:** Bjoern Vogt, Jana Fresen, Georg Gosheger, Harpreet Chhina, Carolin Sophie Brune, Gregor Toporowski, Adrien Frommer, Andrea Laufer, Anthony Cooper, Robert Roedl, Jan Duedal Rölfing

**Affiliations:** 1 Pediatric Orthopedics, Deformity Reconstruction and Foot Surgery, Muenster University Hospital, 48149 Muenster, Germany; 2 General Orthopedics and Tumor Orthopedics, Muenster University Hospital, 48149 Muenster, Germany; 3 Department of Orthopaedic Surgery, BC Children’s Hospital, Vancouver, BC V6H 3N1, Canada; 4 Department of Orthopaedics, Faculty of Medicine, University of British Columbia, Vancouver, BC V6H 3N1, Canada; 5 Children’s Orthopaedics and Reconstruction, Aarhus University Hospital, 8200 Aarhus, Denmark

**Keywords:** patient-reported outcome measures, health-related quality of life, children, lower limb deformities, qualitative interviews, translation and cultural adaptation

## Abstract

(1) Purpose: Lower limb deformities can have a severe impact on health-related quality of life (HRQL). LIMB-Q Kids is a new patient-reported outcome measure (PROM) aiming to elucidate the experience of 8–18-year-old patients before, during and after treatment, and to measure the different aspects of HRQL. The aim of this study was to translate and culturally adapt LIMB-Q Kids to German. (2) Methods: The International Society for Pharmacoeconomics and Outcomes Research (ISPOR) guidelines were followed. Three forward translations, a backward translation, an expert panel meeting with eight participants, and twenty cognitive debriefing interviews led to the final German version of LIMB-Q Kids. (3) Results: In the forward translations, 4/159 items were difficult to translate, and 2/159 items in the backward translation differed from the original English version. Cognitive debriefing interviews with 20 patients identified 7/159 items that were difficult to comprehend/answer, and 2 of these items were changed. (4) Conclusions: Lower limb deformities can have a great impact on children, and it is important to measure and consider the impact on HRQL. In order to be able to use PROMs in different countries, conceptually equivalent translations and cultural adaptations should be performed in order to ensure comprehensibility. The final German version of LIMB-Q Kids is ready for use in an international field test.

## 1. Introduction

Patient-reported outcome measures (PROMs), in addition to clinical findings, provide valuable information about patients’ views on their treatment outcome. Some outcomes can only be described by patients and not verified or measured otherwise [1]. Nowadays, there is an increasing interest in patients’ views on their treatment outcomes, their symptoms, and their health-related quality of life (HRQL). This allows health care professionals to compare types of treatment and assess the well-being of patients [2].

To obtain comparable results, it is easier and timesaving to translate existing PROMs instead of developing a new one [3]. Most of the time, PROMs are developed in one country, in its respective language, then translated, and culturally adapted as needed. A different approach is to collaborate internationally from the beginning. In this way, data can be used to alter and refine the PROM instrument during its development, ensuring that the final version includes patients’ concerns from different cultures and contexts [4]. Additionally, an international collaboration then creates an international database, which allows for the ability to pool and compare results across different countries and cultures [3].

Lower limb deformities can arise from various etiologies, e.g., idiopathic, congenital, or acquired, due to growth disturbances, traumas, and tumors. Lower limb deformities cover a wide range of conditions such as leg length discrepancy, lower limb deficiency, as well as rotational and angular deformities [5]. There are varying treatment options for lower limb deformities, ranging from conservative approaches to complex surgical procedures [6]. Conservative approaches include insoles, shoe lifts, prostheses, and orthotic treatment while the surgical procedures include temporary or permanent growth arrest, lengthening, and reconstructive surgery or amputation. Lengthening and reconstructive procedures can include an intramedullary nail, an external fixator, or a combination of both [6]. Severe deformities may require amputation. In severe deformities, the decision to start a long reconstructive journey or amputate is difficult and there is a lack of evidence [7,8,9]. Hence, the longitudinal studies informed by PROMs may provide a valuable decision aid in the future. 

For leg length discrepancies, there are no studies published comparing the treatment options to the natural course, which complicates helping families and patients come to an informed decision on whether and when to initiate treatment [6].

Lower limb deformities and the resulting physical limitations, pain, and discomfort may lead to reduced involvement and participation in recreational and leisure activities. For children’s development, it is important that they take part regularly in everyday activities [10]. Their limited participation can lead to problems with physiological, emotional, behavioral, and social adjustment [11,12,13]. The factors that have been identified influencing a child’s participation include their social environment (support from family, parents’ interest in recreational activities, community), their functional abilities, and personal attributes (e.g., social ability, age, and gender) [14,15]. Children’s HRQL can therefore be impacted significantly by lower limb deformities [16,17,18,19]. HRQL can be defined as a multi-dimensional concept measuring a person’s perception of their well-being and their physical and mental health [20,21]. In order to accurately measure concepts such as HRQL, it is important to use the patient’s report as the best source of information [22]. These PROMs have to be both reliable and valid and should ideally be developed specifically for a certain condition. In the past, only generic or parent-reported HRQL instruments have been used to measure the HRQL of children with lower limb deformities [23].

Chhina et al., conducted a systematic review on PROMs used for children with lower limb deformities and after identifying the lack of a valid PROM, the development of LIMB-Q Kids was initiated [4,23]. Qualitative interviews were conducted with children from high-income, low-income, and lower-to-middle-income countries to determine what matters most to children with lower limb deformities [24,25]. Item generation and scale development for the new PROM, LIMB-Q Kids, was performed based on qualitative interviews. A content validation stage involved cognitive debriefing interviews with children with lower limb deformities. The interviews were conducted with clinicians and other healthcare professionals involved in the care of children with lower limb deformities. The items and scales were edited based on these interviews. The current version of LIMB-Q Kids consists of 11 scales with 159 items. The scales include function scales (Physical Function Scale, Social Function Scale, Psychological Function Scale), symptoms scales (Leg/Hip/Knee/Ankle/Foot Symptoms Scale), a Leg-related Distress Scale, an Appearance Scale, and a School Scale. There is one stand-alone item about scars which is only applicable to patients who have scars from their surgeries. Content validity was established for the LIMB-Q Kids [25]. 

This version of LIMB-Q Kids is ready for field testing in a large sample of children with lower limb deformities from various international sites. In order to include German-speaking patients in the international field test of LIMB-Q Kids, the aim of the present study was to translate and culturally adapt LIMB-Q Kids to German.

## 2. Materials and Methods

### Translation and Cultural Adaptation (TCA) Process

Scientific rigor demands strict adherence to TCA guidelines in order to create a translation that is linguistically equivalent and also culturally adapted [1]. This study followed the well-established practice guidelines recommended by the International Society For Pharmacoeconomics and Outcomes Research (ISPOR), including the steps: Preparation, Forward Translation, Reconciliation, Back Translation, Back Translation review, Harmonization, Cognitive Debriefing, Review of Cognitive Debriefing Results and Finalization, Proofreading, and Final Report [26].

Written permission was obtained from the original authors of LIMB-Q Kids and the local Research Ethics Committee: no. 2021-556-fS. Written informed consent was obtained from the parents and all children gave their verbal assent according to German legislation [27]. The three participants who were ≥ 18 years old gave their written consent.

Step 1. Preparation

Meetings with the developers of LIMB-Q Kids (H.C.) were held to discuss the process, the relevant steps, and to clarify the objective and aims of this study. 

Step 2. Forward translations and Reconciliation

Three forward translations were performed independently by two German medical students and a professional translator. All three forward translators spoke German as their mother tongue and were fluent in English. A consensus was reached through reconciliation and discussion between the three forward translators resulting in the German version 1.

Step 3. Back translation

An independent professional translator, Sarah Chalmers, whose mother tongue was English and who was fluent in German, produced the back translation from German into English. Discrepancies between the back translation and the original English version were noted and discussed with the developers of LIMB-Q Kids (H.C.).

Step 4: Expert panel meeting and harmonization

The aim of the expert panel meeting was to identify and resolve any inadequate expressions and concepts in the translation, and to ensure that the instrument itself measured all of the clinically relevant issues from the perspective of the clinicians treating children with lower limb deformities in Germany. The German version of LIMB-Q Kids was sent to all of the participants before the meeting. The feedback was used to revise the German translation of LIMB-Q Kids.

Step 5: Cognitive Debriefing Interviews (CDI)

Sampling: In order to include a high variation of diagnoses, age, sex, stage of treatment, and treatment type, purposive sampling was used. ISPOR guidelines recommend to include 5–8 patients in CDI [26]. We decided to include 20 patients to make sure that all age groups and various etiologies for limb differences were represented. 

Patients with lower limb deformities were included, if they were between 8 and 18 years old and were fluent German speakers. The exclusion criteria were: communication problems including not being fluent in German, cognitive impairment, and medical conditions other than limb differences that could impair HRQL. 

Interviews: Using the revised German version, CDIs were performed with patients with limb deformities to ensure that the meaning of the items, response options, and instructions were easy to understand. This was especially important as LIMB-Q Kids is developed as a self-report appropriate for patients between the ages of 8–18 years. 

All of the CDIs were conducted by one of the forward translators (J.F.). The interviewer was trained in the interview methodology by the developer of LIMB-Q Kids (H.C.). In brief, the patients were asked to answer the PROM and mark any text (instruction, item, response option) that was difficult to comprehend or difficult to answer. The interviewer and individual respondent thoroughly went through the entire PROM with special focus on the marked, problematic items. If possible, suggestions for rewording/rephrasing were made. 

Step 6: Review of CDI Results, Finalization, Proofreading, and Final Report

The results from the CDIs were discussed with the LIMB-Q Kids developer (H.C.), and changes to the translation were made as needed. All of the comments were discussed within the expert panel, and the PROM was finalized and proofread, which led to the final German version. 

## 3. Results

Following the ISPOR guidelines gave a rigid framework to and detailed documentation of each of the TCA process steps. Table S1: “*Items that were changed during the TCA process”* provides an overview of all of the challenges and changes during the process.

### 3.1. Forward Translation

The findings from the forward translation showed that the three German translators had different interpretations of the language, which required discussion to reach consensus. Generally, the use of formal versus informal language to address the targeted patients had to be discussed. Formal language is used for professional or academic purposes whereas informal language is used for verbal communication and is more personal. Because LIMB-Q Kids is aimed at patients between 8 and 18 years of age, informal language, e.g., “Du” (informal “you”) instead of “Sie” (formal “you”), and simple wording were chosen in order to make the items easier to read and understand. 

However, a few items were difficult to translate literally, due to different English words with similar meanings that translate to the same German word. For example, the words “swollen” and “puffy” both translate to “geschwollen”, and therefore only one word was used in the German translation.

The item, “My hip/knee/ankle/foot gets stuck (cannot move it)”, provided difficulties during multiple steps of the TCA. Neither the German health care practitioners nor the patients typically use this or a similar expression to describe symptoms. The three forward translations of this item differed in length and complexity. 

One item discussed was, “I forget about my leg when I am at school”. In German, “to forget” translates to “vergessen”, but “to forget about” translates to “nicht an etwas denken” (literal translation: “not thinking about something”). The latter version was chosen to keep the original meaning. 

In total, 4/159 items (2.5%) were difficult to translate and discussed in a consensus meeting before the next step was initiated.

### 3.2. Backward Translation

By comparing the back translation of the German version with the original English version of LIMB-Q Kids, the developers identified a few items whose meaning differed slightly from the original English version or whose reading level seemed higher than the original. These items were discussed in the expert panel meeting.

### 3.3. Expert Panel Meeting

An expert panel meeting was held with the following participants (n = 8): three forward translators, the back translator, a LIMB-Q Kids developer, two German clinicians, and a team member involved in the Danish translation of LIMB-Q Kids. The forward translators and the surgeons spoke German as their mother tongue and were fluent in English, the back translator spoke English as her mother tongue and was fluent in German, and the developer spoke English as her first language. The meeting was held in English. 

In total, the back translation identified 2/159 items (1.3%) whose meaning differed from the English version, which resulted in one item being changed and thus completed the German version ready to be tested on patients.

### 3.4. Cognitive Debriefing Interviews (CDIs) with German Patients

Twenty children with a mean age of 13.9 years (ranging from 9 to 19 years old), with different limb deformities and varying treatments and treatment stages were interviewed. Details on demographic data are presented in Table 1.

In general, the patients found the phrasing and wording of the questionnaire easy to understand and had no major problems filling it out.

One patient noted that using the past week as a time frame (“Please answer thinking of the past week”/“Beziehe Dich bei der Beantwortung bitte auf die letzte Woche”) is too short of a time frame for some of the questions as they might not have tried all of the activities during the past week. 

Additionally, slight difficulties were reported: Two of the patients had not yet tried to sit cross-legged as they had recently undergone surgery and could therefore not answer the question. Two patients were using crutches and did not know whether this circumstance was included in the description, “If you wear a special shoe, shoe-lift or a brace or have an artificial leg (prosthetic leg), please answer the questions thinking of when you have them on”.

At least one or more participants reported difficulty understanding 7/159 (4.4%) items which resulted in two items being changed. Notably, the plain and informal language was well understood even by the vast majority of younger patients.

## 4. Discussion

Following the scientific rigor of the ISPOR guidelines, the present study resulted in a translated and culturally adapted German version of LIMB-Q Kids. The German version is now ready to be part of the ongoing international field test of the PROM assessing the HRQL of 8–18-year-old patients with lower limb deformities. 

PROMs measuring HRQL are on the rise and play a pivotal part in patient information, as well as managing and aligning expectations [27]. Validated PROMs can help to shed light on the natural history of conditions and how these conditions affect HRQL. Furthermore, health care is changing to truly patient-centered care. Collecting information about the effects of various treatment modalities on HRQL is thus important in order to inform patients and their next of kin. PROMs are indispensable in modern health care, because, if developed and validated in an appropriate way, they not only include the patients, but genuinely put them at the center, and measure what is important to them [4,23,24,25,28,29]. PROMs capture more important aspects than mere radiological or other objectively measured outcomes, e.g., joint range of motion, muscle strength, etc. [24,29,30]. The value of PROMs is highlighted by the fact that PROMs do not necessarily correlate well with radiological or other objectively assessed outcomes [31]. 

### 4.1. PROMs for Limb Deformities

A systematic review from 2016 identified a lack of fully developed and validated PROMs for measuring the quality of life of children with lower limb deformities [23].

In adults, Leggett and co-workers are currently developing a PROM for limb deformity patients, called the PROLLIT study. Similar to the approach by the LIMB-Q Kids developers, Leggett et al., conducted a review to explore “what is important to patients with regards to quality of life after experiencing a lower limb reconstructive procedure” [29]. In comparison to LIMB-Q Kids, similar factors, such as ”pain”, “daily functioning” and “emotional wellbeing” can be found, but also additional domains, such as “income” being more relevant to adults. A limitation of PROLLIT is the very specific inclusion criteria of “adults requiring, undergoing or following reconstructive surgery”, which excludes both children and other treatment options [29].

Other limb deformity PROMs are treatment-specific, i.e., the Stanmore Limb Reconstruction Score (SLRS) was developed for patients with “circular ring external fixation devices” [32]. Nonetheless, similar domains, such as “pain”, “mobility”, “physical function”, and “feelings/emotions” can be found with additional scales concerning “sleep” or “hygiene”. Face validity has been documented, however, further testing with a larger group of patients is required to ensure reliability and criterion validity [33].

By adjusting the existing Scoliosis Research Society (SRS) questionnaire for spinal deformities to encompass limb deformities, Fabricant et al., created the LD-SRS [34]. This PROM has since been validated and used for adults with lower limb deformities [30,31,32]. It contains similar scales to LIMB-Q Kids (namely “functioning/activity”, “pain”, or “mental health”) but is not specific to a certain patient age or diagnosis. Depending on whether treatment is planned/ongoing or finished, the PROM only consists of 24 or 30 items, respectively. The LD-SRS is also available in German [30]. Further research is needed to compare the LD-SRS to LIMB-Q Kids and other PROMs for children.

Birch et al., developed a child version from a parent-reported version of the Limb-Lengthening Satisfaction Questionnaire (LLSQ), a questionnaire for parents to assess satisfaction with prostheses in children with limb deficiencies [8,35]. However, it was not reported how the child version was developed and validated [25].

Future studies and field tests are needed to further develop, validate, and compare the PROMs.

### 4.2. Strengths and Limitations

The greatest strength of the present study was in following the rigorous guidelines by the ISPOR combined with an expert panel meeting to find solutions for discrepancies and ensure that the translated items were clinically relevant [26]. It is vital to follow a scientifically proven and valid method to translate a PROM in order to achieve conceptually equivalent items, instructions, and response options. Holding expert panel meetings with the translators and clinical professionals present was important, as the wording used by clinicians often differs from the spoken language of patients and non-healthcare professionals [34]. Information from both parties was found to be complementary to each other, resulting in phrasing that was both medically correct and easy to understand by children.

We performed 20 interviews instead of only 5–8 interviews, as recommended by the ISPOR, in order to take into account, the large variety of types of limb deformities, ages, types of treatment, and treatment stages. By conducting cognitive debriefing interviews, content validity was achieved, and feedback from patients improved the comprehensibility and readability of LIMB-Q Kids. This resulted in a low-grade reading level, which is especially important for PROMs that are supposed to be answered by children themselves. All 20 interviews were conducted by the same person (J.F.), ensuring a consistent methodology.

The major limitation of LIMB-Q Kids is the length of the PROM with 159 items. This might lead to reduced concentration and willingness to read each question thoroughly towards the end of the questionnaire. The upcoming international field test and psychometric testing will likely lead to further item reduction. As noted during the forward translation and pointed out by a few participants during the interviews, several items have similar wording and cover similar areas. However, we anticipate the item reduction phase to identify the best items based on the Rasch analysis. As each sub-scale of LIMB-Q Kids functions independently, there is a flexibility in selecting sub-scales based on clinical and/or research needs [25].

Another limitation of LIMB-Q Kids is the patients’ age. Lower limb deformities are likely to affect a patient’s whole life and not only their childhood. Other PROMs might be more applicable in adults, because they are either specifically designed for this age group, such as PROLLIT, or might be usable for patients of all ages. Future research and the field test will show whether LIMB-Q Kids is compatible with PROMs for adults or can be adapted as the patients grow older. 

The LIMB-Q Kids was developed to cover many different types of lower limb deformities. Even though this study included more patients in interviews than the ISPOR recommends, not all pathologies, diagnoses, and types of treatment were represented. Again, the upcoming international field test, which will include a larger number and variety of patients from around the world, will determine whether LIMB-Q Kids is applicable for all lower limb deformities.

The major strength of LIMB-Q Kids is the patient involvement and rigor during the development. Performing the TCA process in order to be an international project from the beginning and conducting international field tests ensures its relevance. Obtaining input from various linguistic and cultural backgrounds before reaching a finalized version provided the possibility to adjust the original English version to be more generally applicable and transferable [36]. During the upcoming international field test, the inclusion of low-income countries is planned to further increase the general applicability and transferability [25].

## 5. Conclusions

LIMB-Q Kids is now available in German after a rigorous TCA process according to the ISPOR guidelines. This German version is ready to be used in the international field test (Figure S1). Field test data will be used to perform item reduction and psychometric testing of LIMB-Q Kids.

Ultimately, LIMB-Q Kids could be used to evaluate the conditions and interventions in regard to symptoms, leg appearance, and general HRQL in children with lower limb deformities. By comparing the HRQL, healthcare professionals will be able to help patients and their families come to informed decisions about different treatment plans and interventions [25], such as the choice between reconstruction and amputation [4].

## Figures and Tables

**Table 1:** Demographic data: patient’s age at the time of the interview, sex, diagnosed limb deformity, treatment, and treatment stage.

Patient	Age	Sex *^1^	Diagnosis *^2^	Treatment *^3^	Stage of Treatment
1	12.2	F	Post-traumatic LLD 4.4 cm	Lengthening nail	Consolidation period
2	10.6	F	Congenital LLD (CFD)	Femoropelvic fusion	Prosthetic treatment
3	16.8	F	Post-infectious LLD 11.5 cm (neonatal osteomyelitis)	External ring fixator → trauma nail	Consolidation period
4	16.3	M	Idiopathic Genu varum	High tibial osteotomy, plate fixation	Newly operated
5	13.6	M	Post-infectious LLD 5.0 cm and genu valgum (Neonatal septic arthritis distal femur)	Planned gradual lengthening	Not yet treated
6	14.5	F	Congenital LLD 3.0 cm, posteromedial tibial bowing	Lengthening nail	Consolidation period
7	18.0	M	Post-tumor LLD 5.2 cm (resected sarcoma)	Lengthening nail	Distraction period
8	19.9	M	Disproportionate short stature, Genua vara (SHOX (short stature homeobox) gene mutation)	Lengthening nail	Distraction period
9	14.8	M	Idiopathic Genua valga	Guided growth	Devices in situ
10	11.8	F	Idiopathic flexible flat feet	Calcaneo-stop procedure	Devices in situ
11	11.5	F	Congenital LLD 7.0 cm (CFD)	Lengthening nail (infection) → external fixator (goal 4–5 cm)	Distraction period
12	17.9	M	Idiopathic LLD 3.5 cm	Lengthening nail	Distraction period
13	14.4	M	Idiopathic Genua valga	Guided growth	After removal
14	16.4	M	Idiopathic Genua valga	Guided growth	After removal
15	9.3	M	Disproportionate short stature, genu varum (achondroplasia)	Guided growth	Before treatment
16	9.1	M	Congenital LLD > 5.0 cm, CFD	Intramedullary lengthening nail	Before treatment
17	11.4	F	Congenital LLD 4.0 cm, ankle valgus (fibular hemimelia)	SHORDT procedure (SHortening Osteotomy Realignment Distal Tibia)	Consolidation period
18	11.6	F	CFD, fibular hemimelia, genu valgum	Guided growth, external fixation, ACL reconstruction	After treatment
19	9.4	F	Idiopathic tall stature (final body height 192 cm)	Planned permanent growth arrest	Before treatment
20	19.0	F	Disproportionate short stature (Turner syndrome)	Lengthening nail	Distraction period

*^1^ M: male; F: female; *^2^ LLD: leg length discrepancy; CFD: congenital femoral deficiency; *^3^ ACL: anterior cruciate ligament.

## Data Availability

Data can be requested from the corresponding author, bjoern.vogt@ukmuenster.de.

## Supplementary Materials

The following supporting information can be downloaded at: https://www.mdpi.com/article/10.3390/children9091405/s1, Table S1: Items that were changed during the TCA process; Figure S1: The German version of LIMB-Q Kids.
