# Supplementary material for: LIMB-Q Kids—German Translation and Cultural Adaptation
Source: Children (Basel). 2022 Sep 16;9(9):1405. doi: 10.3390/children9091405 (PMC9498267; doi:10.3390/children9091405)
Supplement: Supplementary file 1 [file children-09-01405-s001.zip › Figure S1 German Field Test Version of LIMB-Q-Kids.pdf]

LIMB-Q Kids Fragebogen

Funktion Bein

Wie leicht fällt es Dir, Dein Bein/ Deine Beine zu benutzen?
Beantworte jede Frage, indem Du eine Zahl umkreist. Beziehe Dich bei der Beantwortung bitte auf die letzte Woche. Die Fragen beziehen sich auf das Bein, weswegen Du zum Arzt gehst.

Wenn Du einen Spezialschuh, eine Schuherhöhung oder eine Schiene trägst oder ein künstliches Bein (Beinprothese) hast, denk bitte beim Beantworten der Fragen an Situationen, in denen Du diese(n) trägst.

Kannst Du:	Das KANN ich NICHT	Das fällt mir SEHR SCHWER	Das fällt mir EIN WENIG SCHWER	Das KANN ich
1. ...aus dem Bett aufstehen?	1	2	3	4
2. ...auf einem Stuhl sitzen?	1	2	3	4
3. ...von einem Stuhl aufstehen?	1	2	3	4
4. ...auf der Toilette sitzen?	1	2	3	4
5. ...im Schneidersitz auf dem Boden sitzen?	1	2	3	4
6. ...vom Boden aufstehen?	1	2	3	4
7. ...ein Bein heben, während Du auf dem anderen stehst?	1	2	3	4
8. ...Deine Knie beugen?	1	2	3	4
9. ...ein Buch vom Boden aufheben?	1	2	3	4
10. ...gehen, ohne zu humpeln?	1	2	3	4
11. ...gehen, ohne hinzufallen?	1	2	3	4
12. ...auf holprigem Untergrund gehen?	1	2	3	4
13. ...so schnell gehen, wie du möchtest?	1	2	3	4
14. ...so weit gehen, wie du möchtest?	1	2	3	4
15. ...Treppen hochgehen?	1	2	3	4
16. ...Treppen runtergehen?	1	2	3	4
17. ...beim Gehen ein Buch tragen?	1	2	3	4
18. ...bergaufgehen?	1	2	3	4
19. ...bergabgehen?	1	2	3	4
20. ...für längere Zeit stehen?	1	2	3	4
21. ...klettern (zum Beispiel eine Leiter hoch)?	1	2	3	4
22. ...rennen?	1	2	3	4
23. ...rennen, ohne hinzufallen?	1	2	3	4
24. ...so schnell rennen, wie Du möchtest?	1	2	3	4
25. ...so weit rennen, wie Du möchtest?	1	2	3	4
26. ...Fahrrad fahren?	1	2	3	4
27. ...Sport machen, den Du magst?	1	2	3	4
28. ...springen?	1	2	3	4
29. ...Schuhe an- oder ausziehen?	1	2	3	4
30. ...Kleidung an- oder ausziehen?	1	2	3	4
31. ...auf einem Bein hüpfen?	1	2	3	4
32. ...auf einem Bein stehen?	1	2	3	4

Beschwerden Bein

Wie fühlt sich Dein Bein an? Beantworte jede Frage, indem Du eine Zahl umkreist. Beziehe Dich bei der Beantwortung bitte auf die letzte Woche.

Die Fragen beziehen sich auf das Bein, weswegen Du zum Arzt gehst.

	Nie	Manchmal	Oft	Immer
1. Mein Bein schmerzt, wenn ich mich ausruhe oder entspanne.	1	2	3	4
2. Mein Bein schmerzt, wenn ich schlafe.	1	2	3	4
3. Mein Bein schmerzt, wenn ich längere Zeit stehe.	1	2	3	4
4. Mein Bein schmerzt, wenn ich gehe.	1	2	3	4
5. Mein Bein schmerzt, wenn ich renne.	1	2	3	4
6. Mein Bein schmerzt, wenn ich es berühre.	1	2	3	4
7. Mein Bein ermüdet, wenn ich gehe.	1	2	3	4
8. Mein Bein ermüdet, wenn ich renne.	1	2	3	4
9. Mein Bein fühlt sich schwach (wackelig) an.	1	2	3	4
10. Mein Bein fühlt sich taub an (ich kann es nicht spüren).	1	2	3	4
11. Mein Bein fühlt sich kribbelig an (wie Nadelstiche).	1	2	3	4
12. Mein Bein ist geschwollen.	1	2	3	4
13. Mein Bein juckt.	1	2	3	4
14. Ich habe einen Ausschlag am Bein (Rötung).	1	2	3	4

Beschwerden Hüfte

Wie fühlt sich Deine Hüfte an? Beantworte jede Frage, indem Du eine Zahl umkreist. Beziehe Dich bei der Beantwortung bitte auf die letzte Woche.

Die Fragen beziehen sich auf die Hüfte des Beines, weswegen Du zum Arzt gehst.

	Nie	Manchmal	Oft	Immer
1. Meine Hüfte schmerzt, wenn ich mich ausruhe oder entspanne.	1	2	3	4
2. Meine Hüfte schmerzt, wenn ich längere Zeit sitze.	1	2	3	4
3. Meine Hüfte schmerzt, wenn ich längere Zeit stehe.	1	2	3	4
4. Meine Hüfte schmerzt, wenn ich gehe.	1	2	3	4
5. Meine Hüfte schmerzt, wenn ich Treppen hinaufsteige.	1	2	3	4
6. Meine Hüfte schmerzt, wenn ich Treppen hinabsteige.	1	2	3	4
7. Meine Hüfte schmerzt, wenn ich renne.	1	2	3	4
8. Meine Hüfte schmerzt, wenn sie ein Geräusch macht (Klicken oder Knacken).	1	2	3	4
9. Meine Hüfte blockiert (ich kann sie nicht bewegen).	1	2	3	4
10. Meine Hüfte fühlt sich schwach an (wackelig).	1	2	3	4

Beschwerden Knie

Wie fühlt sich Dein Knie an? Beantworte jede Frage, indem Du eine Zahl umkreist. Beziehe Dich bei der Beantwortung bitte auf die letzte Woche.

Die Fragen beziehen sich auf das Knie des Beines, weswegen Du zum Arzt gehst.

	Nie	Manchmal	Oft	Immer
1. Mein Knie schmerzt, wenn ich mich ausruhe oder entspanne.	1	2	3	4
2. Mein Knie schmerzt, wenn ich längere Zeit stehe.	1	2	3	4
3. Mein Knie schmerzt, wenn ich gehe.	1	2	3	4
4. Mein Knie schmerzt, wenn ich Treppen hinaufsteige.	1	2	3	4
5. Mein Knie schmerzt, wenn ich Treppen hinabsteige.	1	2	3	4
6. Mein Knie schmerzt, wenn ich renne.	1	2	3	4
7. Mein Knie schmerzt, wenn ich es beuge.	1	2	3	4
8. Mein Knie schmerzt, wenn ich es ausstrecke.	1	2	3	4
9. Mein Knie schmerzt, wenn es ein Geräusch macht (Klicken oder Knacken).	1	2	3	4
10. Mein Knie blockiert (ich kann es nicht bewegen).	1	2	3	4
11. Mein Knie fühlt sich unsicher an (als ob es wegknickt).	1	2	3	4
12. Mein Knie fühlt sich schwach an (wackelig).	1	2	3	4
13. Mein Knie ist geschwollen.	1	2	3	4

Beschwerden Sprunggelenk

Wie fühlt sich Dein Sprunggelenk an? Beantworte jede Frage, indem Du eine Zahl umkreist. Beziehe Dich bei der Beantwortung bitte auf die letzte Woche.

Die Fragen beziehen sich auf das Sprunggelenk des Beines, weswegen Du zum Arzt gehst.

	Nie	Manchmal	Oft	Immer
1. Mein Sprunggelenk schmerzt, wenn ich mich ausruhe oder entspanne.	1	2	3	4
2. Mein Sprunggelenk schmerzt, wenn ich längere Zeit stehe.	1	2	3	4
3. Mein Sprunggelenk schmerzt, wenn ich gehe.	1	2	3	4
4. Mein Sprunggelenk schmerzt, wenn ich auf holprigem Untergrund gehe.	1	2	3	4
5. Mein Sprunggelenk schmerzt, wenn ich renne.	1	2	3	4
6. Mein Sprunggelenk schmerzt, wenn es ein Geräusch macht (Klicken oder Knacken).	1	2	3	4
7. Mein Sprunggelenk blockiert (ich kann es nicht bewegen).	1	2	3	4
8. Mein Sprunggelenk fühlt sich unsicher an (als ob es wegknickt).	1	2	3	4
9. Mein Sprunggelenk fühlt sich schwach an (wackelig).	1	2	3	4
10. Mein Sprunggelenk ist geschwollen.	1	2	3	4

Beschwerden Fuß

Wie fühlt sich Dein Fuß an? Beantworte jede Frage, indem Du eine Zahl umkreist. Beziehe Dich bei der Beantwortung bitte auf die letzte Woche.

Die Fragen beziehen sich auf den Fuß des Beines, weswegen Du zum Arzt gehst.

	Nie	Manchmal	Oft	Immer
1. Mein Fuß schmerzt, wenn ich mich ausruhe oder entspanne.	1	2	3	4
2. Mein Fuß schmerzt, wenn ich längere Zeit stehe.	1	2	3	4
3. Mein Fuß schmerzt, wenn ich auf holprigem Untergrund gehe.	1	2	3	4
4. Mein Fuß schmerzt, wenn ich renne.	1	2	3	4
5. Mein Fuß schmerzt, wenn ich mit Schuhen gehe.	1	2	3	4
6. Mein Fuß schmerzt, wenn ich barfuß gehe (ohne Schuhe).	1	2	3	4
7. Mein Fuß blockiert (ich kann ihn nicht bewegen).	1	2	3	4
8. Mein Fuß fühlt sich schwach an (wackelig).	1	2	3	4
9. Mein Fuß ist geschwollen.	1	2	3	4

Empfinden Bein

Wie geht es Dir mit Deinem Bein? Beantworte jede Frage, indem Du eine Zahl umkreist. Beziehe Dich bei der Beantwortung bitte auf die letzte Woche.
Die Fragen beziehen sich auf das Bein, weswegen Du zum Arzt gehst.

Wenn Du einen Spezialschuh, eine Schuherhöhung oder eine Schiene trägst oder ein künstliches Bein (Beinprothese) hast, denk bitte beim Beantworten der Fragen an Situationen, in denen Du diese(n) trägst.

	Nie	Manchmal	Oft	Immer
1. Ich verstecke mein Bein, wenn ich rausgehe.	1	2	3	4
2. Ich vermeide es, kurze Hosen oder Röcke zu tragen, die mein Bein zeigen.	1	2	3	4
3. Ich mag es nicht, wie mein Bein aussieht.	1	2	3	4
4. Ich bin unglücklich über mein Bein.	1	2	3	4
5. Ich mache mir sorgen um mein Bein.	1	2	3	4
6. Es stört mich, wenn Leute mein Bein anschauen.	1	2	3	4
7. Es stört mich, wenn mich Leute nach meinem Bein fragen.	1	2	3	4
8. Ich mag keine Fotos, die mein Bein zeigen.	1	2	3	4
9. Ich vermeide es aufgrund meines Beines auszugehen (zum Beispiel zu einer Party).	1	2	3	4
10. Ich mag es nicht, wie ich aussehe, wenn ich gehe.	1	2	3	4
11. Durch mein Bein fühle ich mich anders als andere Leute.	1	2	3	4
12. Ich werde aufgrund meines Beines geärgert.	1	2	3	4
13. Es stört mich, wenn ich wegen meines Beines keinen Spaß haben kann.	1	2	3	4
14. Es stört mich, wenn ich nicht die Art von Schuhen tragen kann, die ich mag.	1	2	3	4

Aussehen Bein

Wie sieht Dein Bein aus? Beantworte jede Frage, indem Du eine Zahl umkreist. Beziehe Dich bei der Beantwortung bitte auf die letzte Woche.
Die Fragen beziehen sich auf das Bein, weswegen Du zum Arzt gehst.

Wenn Du einen Spezialschuh, eine Schuherhöhung oder eine Schiene trägst oder ein künstliches Bein (Beinprothese) hast, denk bitte beim Beantworten der Fragen an Situationen, in denen Du diese(n) trägst.

Wie sehr magst du…	Überhaupt nicht	Ein bisschen	Ziemlich	Sehr
1. ...wie Dein Bein aussieht, wenn du gehst?	1	2	3	4
2. ...wie Dein Bein aussieht, wenn du rennst?	1	2	3	4
3. ...die Länge Deines Beines?	1	2	3	4
4. ...den Umfang/Größe Deines Beines?	1	2	3	4
5. ...wie Dein Bein aussieht, wenn Du eine kurze Hose oder einen Rock trägst?	1	2	3	4
6. ...wie Dein Bein aussieht, wenn Du eine lange Hose oder eine Jeans trägst?	1	2	3	4
7. ...wie Dein Bein auf Fotos aussieht?	1	2	3	4
8. ...wie Dein Bein aussieht, wenn Du stehst?	1	2	3	4
9. ...wie Dein Bein aussieht, wenn Du auf dem Boden sitzt?	1	2	3	4
10. ...wie Dein Bein aussieht, wenn Du auf einem Stuhl sitzt?	1	2	3	4
11. ...wie Dein Bein im Vergleich zu den Beinen von anderen Leuten aussieht?	1	2	3	4
12. ...wie gut Deine Beine zueinander passen (gleich aussehen)?	1	2	3	4
13. ...wie gerade Dein Bein aussieht?	1	2	3	4
14. ...wie Dein Fuß aussieht?	1	2	3	4
15. ...wie Dein Knie aussieht?	1	2	3	4
16. ...wie gut Deine Hüften zueinander passen (gerade oder schief)?	1	2	3	4
17. ...wie Dein Bein insgesamt aussieht?	1	2	3	4

Narben **(für Patienten, die bereits operiert wurden)**

Hast du Narben von Deiner/Deinen Bein-Operation(en)? Falls ja, beantworte jede Frage, indem Du eine Zahl umkreist. Beziehe Dich bei der Beantwortung bitte darauf, wie deine Narben jetzt aussehen.
Die Fragen beziehen sich auf das Bein, weswegen Du zum Arzt gehst.

Wie sehr magst du…	Überhaupt nicht	Ein bisschen	Ziemlich	Sehr
1. ...wie die Narben auf Deinem Bein aussehen?	1	2	3	4

Schule

Wie geht es Dir in der Schule? Beantworte jede Frage, indem Du eine Zahl umkreist. Beziehe Dich bei der Beantwortung bitte auf die letzte Woche.

HINWEIS: Wenn Du in der letzten Woche nicht in der Schule warst, denke bitte an die Zeit, in der Du zuletzt in der Schule warst.

	Nie	Manchmal	Oft	Immer
1. Ich mag es, meine Freunde in der Schule zu sehen.	1	2	3	4
2. Meine Lehrer helfen mir in der Schule.	1	2	3	4
3. Ich fühle mich in der Schule akzeptiert.	1	2	3	4
4. Andere Schüler mögen mich.	1	2	3	4
5. Ich bin glücklich in der Schule.	1	2	3	4
6. Andere Schüler sind nett zu mir.	1	2	3	4
7. Andere Schüler hören mir zu, wenn ich rede.	1	2	3	4
8. Andere Schüler helfen mir in der Schule.	1	2	3	4
9. Ich fühle mich sicher in der Schule (kein Mobbing).	1	2	3	4
10. Es fällt mir leicht, neue Freunde zu finden.	1	2	3	4
11. Ich werde gefragt, ob ich bei schulischen Aktivitäten oder Spielen mitmachen möchte.	1	2	3	4
12. Ich fühle mich in der Schule zugehörig.	1	2	3	4
13. Ich gehe gerne zur Schule.	1	2	3	4
14. Es fällt mir leicht, in der Schule zum Unterricht zu gelangen.	1	2	3	4
15. Ich vergesse mein Bein, wenn ich in der Schule bin.	1	2	3	4
16. Es ist in Ordnung für mich, wenn andere Schüler mich nach meinem Bein fragen.	1	2	3	4
17. Meine Lehrer behandeln mich gut.	1	2	3	4

Sozialleben

Wie ist Dein Sozialleben? Beantworte jede Frage, indem Du eine Zahl umkreist. Beziehe Dich bei der Beantwortung bitte auf die letzte Woche.

	Nie	Manchmal	Oft	Immer
1. Meine Freunde akzeptieren mich.	1	2	3	4
2. Ich habe Spaß mit Freunden.	1	2	3	4
3. Personen hören mir zu, wenn ich etwas sagen möchte.	1	2	3	4
4. Personen behandeln mich genauso wie alle anderen auch.	1	2	3	4
5. Ich bin gerne mit anderen Leuten zusammen.	1	2	3	4
6. Ich fühle mich selbstbewusst, wenn ich ausgehe (zum Beispiel zu einer Party).	1	2	3	4
7. Ich werde gefragt, ob ich mit Freunden ausgehen möchte (zum Beispiel zu einer Party).	1	2	3	4
8. Ich fühle mich zugehörig.	1	2	3	4
9. Es fällt mir leicht, neue Freunde zu finden.	1	2	3	4
10. Ich fühle mich genauso wie andere Leute in meinem Alter.	1	2	3	4
11. Es ist in Ordnung, wenn andere Leute mein Bein anschauen.	1	2	3	4
12. Es ist in Ordnung, wenn mich andere Leute nach meinem Bein fragen.	1	2	3	4

Empfinden

Wie fühlst Du Dich? Beantworte jede Frage, indem Du eine Zahl umkreist. Beziehe Dich bei der Beantwortung bitte auf die letzte Woche.

	Nie	Manchmal	Oft	Immer
1. Ich bin glücklich mit meinem Leben.	1	2	3	4
2. Ich genieße das Leben.	1	2	3	4
3. Ich fühle mich glücklich.	1	2	3	4
4. Ich finde mich okay, so wie ich bin.	1	2	3	4
5. Ich glaube an mich selbst.	1	2	3	4
6. Ich bin stolz auf mich.	1	2	3	4
7. Ich mag mich selbst.	1	2	3	4
8. Ich fühle mich selbstbewusst.	1	2	3	4
9. Ich finde mich toll, so wie ich bin.	1	2	3	4
10. Ich bin mit meinem Aussehen zufrieden.	1	2	3	4
