# Supplementary material for: LIMB-Q Kids—German Translation and Cultural Adaptation
Source: Children (Basel). 2022 Sep 16;9(9):1405. doi: 10.3390/children9091405 (PMC9498267; doi:10.3390/children9091405)
Supplement: Supplementary file 1 [file children-09-01405-s001.zip › Table S1 Items that were changed during the TCA process.pdf]

Table S1: Items that were changed during the TCA process

Scale	Item	Problem Forward Translation	Backward Translation	Decision/Changes after Expert Panel Meeting	Problem Cognitive Debriefing Interviews	Solution after Cognitive Debriefing Interviews
Use legs	These questions ask about the leg you are seeing the doctor or nurse about.	In Germany nurses do not have the same field of work and authority as nurses in some English-speaking countries and a patient is always going to see a doctor at every visit.	no problem	It was decided to only translate doctor.	no problem, no changes	
Use legs	Are you able to:	no problem	Can you:	The words "to be able to" and "to can" have the same meaning (= können), therefore the meaning is retained.	no problem, no changes	
Use legs	5. …sit cross legged on the floor?	discussed "Schneidersitz" vs. "mit überkreuzten Beinen"	no problem	Decided on "Schneidersitz" as it is shorter and just as easy to understand.	two participants had not tried sitting cross-legged due to recent operation, one patient did not know the word "Schneidersitz"	noted, no change
Use legs	10. …walk without a limp?	no problem	10. …walk without limping?	original item walk without a limp has a lower reading level but a child should understand the German version.	no problem, no changes	
Use legs	12. …walk on a bumpy road?	Discussion whether to find a less literal translation for "road" to take other surfaces into account	12. ...walk on a bumpy surface?	Translation "12. ...auf holprigem Untergrund gehen?" in order to include other surfaces.	no problem, no changes	
Leg feel	12. My leg is swollen or puffy.	English original: two adjectives used to describe the phenomenon concerned German translation: both adjectives describe the same feeling "geschwollen"	no problem	It was decided to only use one adjective "geschwollen" in the German version as there are no German words to explain the difference.	no problem, no changes	

Scale	Item	Problem Forward Translation	Backward Translation	Decision/Changes after Expert Panel Meeting	Problem Cognitive Debriefing Interviews	Solution after Cognitive Debriefing Interviews
Leg feel	14. My leg has a rash (looks red).	no problem	no problem	no problem, no change	one patient was confused as their scars were red (but not a rash)	noted, no change
Hip feel	9. My hip gets stuck (not able to move it).	No literal German translation,<br>the symptom of a "stuck hip/knee/ankle/foot" is not one that is typically talked about in German	9. My hip gets blocked (I cannot move it).	Difficult to find an expression which is both accurate and easy to understand for children. Conclusion: item is made clear by the addition in brackets	Four patients did not know what this meant/could not imagine the feeling/did not understand what was asked	problem: patients reply yes but are thinking about swelling or other reasons why they cannot move their hip/knee etc. --> different meaning --> noted, if difficulties in Field Test/other languages as well, might change
Knee feel	11. My knee feels wobbly (like it is going to give out).	"wobbly" and "shaky" translate to similar words in German	no problem	In order to achieve a consistent translation throughout the different scales it was decided to translate "shaky" with "wackelig" and "wobbly" with "unsicher" (literal translation: unsafe).		
Knee feel	12. My knee feels weak (shaky).		no problem		no problem, no changes	
Ankle feel	How does your ankle feel?	Three different possible translations for "ankle" (="Fußgelenk", "Sprunggelenk", "Knöchel")	no problem	Consensus was to use "Sprunggelenk" as it is the anatomically correct expression and also thought to be understandable for children.	two patients did not know the word "Sprunggelenk", one of which also did not know the word "Knöchel" which had been discussed as an alternative in the expert panel meeting	noted, no changes as it is the anatomical correct translation and all other patients understood --> might have to be explained by supervising adult
You feel about your leg	5. I worry about my leg.	no problem	no problem	no problem, no change	Two patients did not know whether "sorgen" refers to "versorgen" (to care about/take care of) or "sich Sorgen machen" (to worry about)	The translation was changed to "5. Ich mache mir Sorgen um mein Bein." to avoid confusion; original meaning retained

Scale	Item	Problem Forward Translation	Backward Translation	Decision/Changes after Expert Panel Meeting	Problem Cognitive Debriefing Interviews	Solution after Cognitive Debriefing Interviews
						(even better than previous version)
You feel about your leg	9. I avoid going out because of my leg (like to a party).	no problem	no problem	no problem, no change	Party does not apply for younger children, some parents/patients suggested something like "Spielplatz" (playground) or "Übernachtungsparty" (sleep over) as an alternative example	noted, no changes --> flagged: if problem occurs in field testing/other languages might change
Leg looks	4. …the size of your leg?	4. ...den Umfang/Größe Deines Beines?	4. ...the circumference/size of your leg?	no change	two patients did not know the word "Umfang" (circumference) and suggested to use "Dicke" (thickness) or "Breite" (wideness) instead	Decision to keep the word "Umfang" as it is closer to the original meaning
School	2. My teachers help me at school.	no problem	no problem	no problem, no change	Three patients did not know whether this referred to physical help regarding their leg or academic help/assistance	The item is asking about the requiring of physical help from the teachers. While this might not be clear to all patients most should know that the questionnaire is about their leg and therefore answer in respect to their physical abilities.
School	12. I fit in at school.	no problem	no problem	no problem, no change	One patient did not know the word "zugehörig" (belonging to)	noted, no changes, as all other wordings would be more difficult to understand / would not make sense

Scale	Item	Problem Forward Translation	Backward Translation	Decision/Changes after Expert Panel Meeting	Problem Cognitive Debriefing Interviews	Solution after Cognitive Debriefing Interviews
School	15. I forget about my leg when I am at school.	The German translation for “Forget about“ in order to retain the meaning would be "I do not think about my leg"	15. I don’t think about my leg when I am at school.		Question includes negation in German (I do not think about my leg) making replying difficult "Nie" (never) and "Immer" (always) means (double negative).	Translation was changed to "15. Ich vergesse mein Bein, wenn ich in der Schule bin." (closer to English original but might sound strange to German participants) --> check during field testing
Feel	1. I am happy with my life.	More common in German to say "Ich bin zufrieden mit meinem Leben" which translates to "being satisfied"	1. I am satisfied with my life.	Difficulty finding word happy in German as items 1 and 3 are very close. Consensus was to keep the same word for 'happy' as used in item 3 to be consistent with the English version and German for item 3	no problem, no changes	
